# Draft Genome Sequence of the White-Rot Fungus *Obba rivulosa* 3A-2

**DOI:** 10.1128/genomeA.00976-16

**Published:** 2016-09-15

**Authors:** Otto Miettinen, Robert Riley, Kerrie Barry, Dan Cullen, Ronald P. de Vries, Matthieu Hainaut, Annele Hatakka, Bernard Henrissat, Kristiina Hildén, Rita Kuo, Kurt LaButti, Anna Lipzen, Miia R. Mäkelä, Laura Sandor, Joseph W. Spatafora, Igor V. Grigoriev, David S. Hibbett

**Affiliations:** a Finnish Museum of Natural History, University of Helsinki, Helsinki, Finland; b U.S. Department of Energy (DOE) Joint Genome Institute, Walnut Creek, California, USA; c U.S. Department of Agriculture (USDA), Forest Service, Forest Products Laboratory, Madison, Wisconsin, USA; d Fungal Physiology, CBS-KNAW Fungal Biodiversity Centre, Utrecht, the Netherlands; e Fungal Molecular Physiology, Utrecht University, the Netherlands; f Division of Microbiology and Biotechnology, Department of Food and Environmental Sciences, University of Helsinki, Helsinki, Finland; g Centre National de la Recherche Scientifique, Aix-Marseille Université, Marseille, France; h INRA, Marseille, France; i Department of Biological Sciences, King Abdulaziz University, Jeddah, Saudi Arabia; j Department of Botany and Plant Pathology, Oregon State University, Corvallis, Oregon, USA; k Department of Biology, Clark University, Worcester, Massachusetts, USA

## Abstract

We report here the first genome sequence of the white-rot fungus *Obba rivulosa* (Polyporales, Basidiomycota), a polypore known for its lignin-decomposing ability. The genome is based on the homokaryon 3A-2 originating in Finland. The genome is typical in size and carbohydrate active enzyme (CAZy) content for wood-decomposing basidiomycetes.

## GENOME ANNOUNCEMENT

*Obba rivulosa* (= *Physisporinus rivulosus*) is a conifer-dwelling polypore, a selective delignifier that targets lignin over cellulose in the early stages of wood decomposition (1, 2). This makes the species a promising object for studies in lignin degradation and offers prospects for the pretreatment of wood in the pulp industry (3) and bioremediation of soils containing chlorinated compounds (4). *Obba rivulosa* is a close relative of *Gelatoporia subvermispora* (*= Ceriporiopsis subvermispora*), whose draft genome sequence has been published (5). *Gelatoporia* and *Obba* belong to the *Gelatoporia* clade, an evolutionarily distinctive group within *Polyporales* (*Basidiomycota*) (6).

North American and Eurasian populations of *O. rivulosa* differ slightly genetically (7). This genome is based on a monokaryon (3A-2) created from a European strain (T241i, FBCC 949) isolated from a charred conifer log in Finland. Its manganese peroxidases and laccases have been characterized (8–11).

The strain was grown in 2% malt broth. DNA was extracted from manually ground mycelium with the DNeasy plant maxi kit and RNA with RNeasy midi kit (Qiagen). Both the genome and transcriptome were sequenced using Illumina platforms. DNA was sheared to 270 bp using the Covaris E210 (Covaris) and size-selected using SPRI beads (Beckman Coulter). The fragments were treated with end repair, A-tailing, and ligation of Illumina adapters using the TruSeq sample prep kit (Illumina). Stranded cDNA libraries were generated using the TruSeq stranded RNA LT kit. mRNA was purified from total RNA using magnetic beads containing poly(T) oligonucleotides, fragmented, and reverse transcribed using random hexamers and SSII (Invitrogen), followed by second-strand synthesis. The fragmented cDNA was treated with end repair, A-tailing, adapter ligation, and eight cycles of PCR. Both libraries were quantified using Kapa Biosystems’ next-generation sequencing library quantitative PCR (qPCR) kit and run on a Roche LightCycler 480 real-time PCR instrument. The quantified library was then prepared for sequencing on the Illumina HiSeq sequencing platform utilizing a TruSeq paired-end cluster kit, version 4, and Illumina’s cBot instrument to generate clustered flow cells for sequencing on the Illumina HiSeq 2500 using HiSeq and TruSeq SBS sequencing kits, version 4, following a 2 × 150 indexed run recipe.

Genomic reads were filtered for contamination and initially assembled with Velvet (12). The resulting assembly was used to create an *in silico* long mate-pair library with 3-kbp insert, which was then assembled with the original Illumina library with AllPaths-LG release 46652 (13) to produce the 34.04-Mbp assembly in 712 scaffolds. The transcriptome was assembled using Rnnotator (14). The genome was annotated using the JGI annotation pipeline, resulting in 13,206 predicted genes.

The assembly length and estimated number of genes are typical of saprobic basidiomycetes. Multiple gene models encoding enzymes implicated in lignin degradation are present (nine manganese peroxidases and nine laccases). A single lignin peroxidase gene, but no versatile peroxidase, was detected. Among *Polyporales*, the carbohydrate active enzyme (CAZy) (15) profile is most similar to that of the closely related *Gelatoporia subvermispora*.

### Accession number(s).

This whole-genome shotgun project has been deposited at DDBJ/ENA/GenBank under the accession no. MAXV00000000. The version described in this paper is MAXV01000000. The annotated genome is available via the JGI fungal genome portal MycoCosm (http://genome.jgi.doe.gov/Obbri1) (16).
